# Supplementary material for: Shared Binding Site but Divergent Resistance Profiles Uncover Novel Resistance Mechanisms in Plasmodium HSP90 Inhibitors
Source: bioRxiv. 2025 Nov 1:2025.10.30.684657. Preprint. [Version 1] doi: 10.1101/2025.10.30.684657 (PMC12636505; doi:10.1101/2025.10.30.684657)
Supplement: Supplement 4 [file NIHPP2025.10.30.684657v1-supplement-4.pdf]

bioRxiv preprint doi: https://doi.org/10.1101/2025.10.30.684657; this version posted November 1, 2025. The copyright holder for this preprint (which was not certified by peer review) is the author/funder, who has granted bioRxiv a license to display the preprint in perpetuity. It is made available under aCC-BY 4.0 International license.

Fig S1

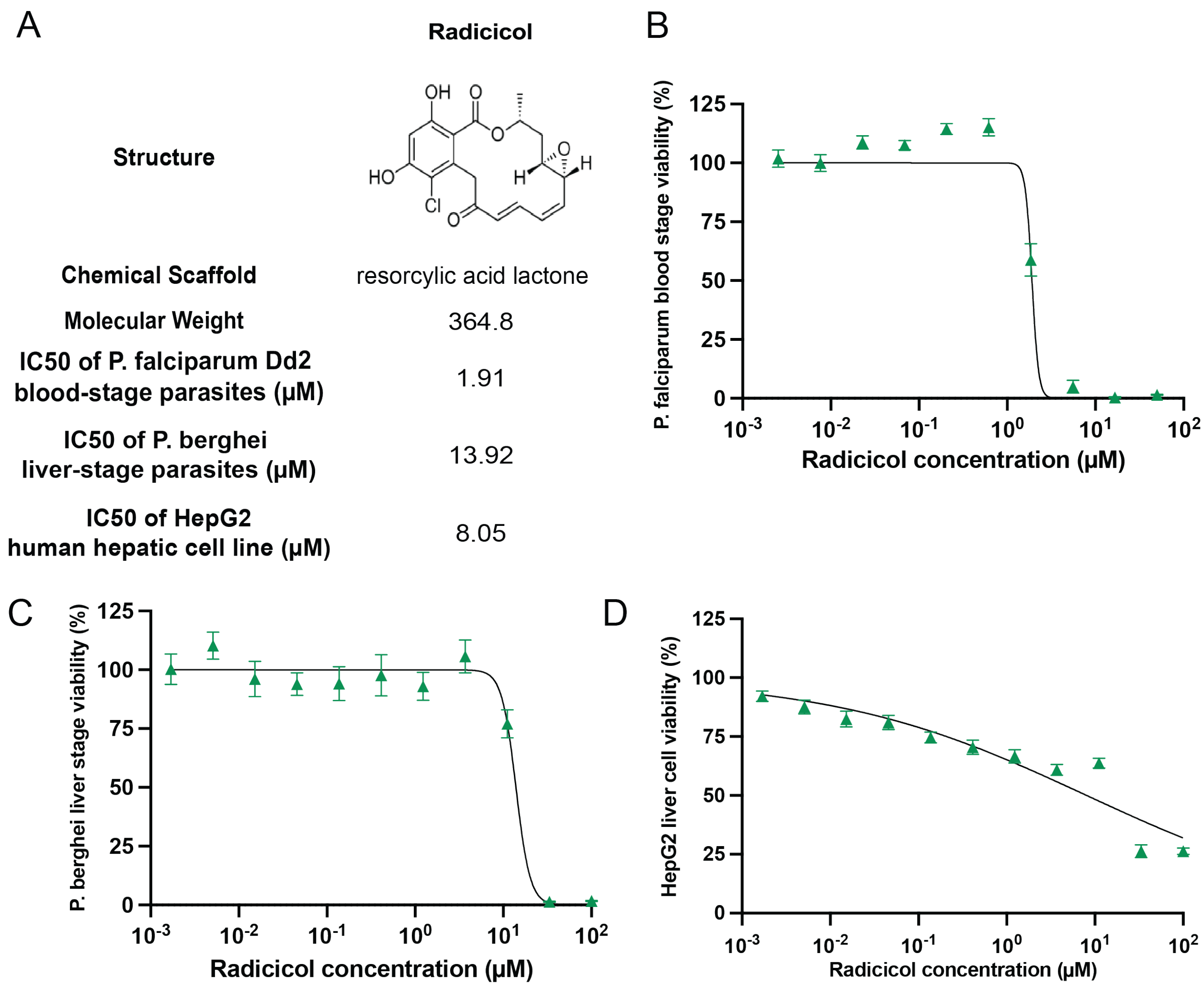

Figure S1. Antimalarial characterization of radicicol as an HSP90 inhibitor

(A) Chemical structure and biological activity profile of radicicol showing its resorcylic acid lactone scaffold and IC_{50} values against different parasite stages and human cells. **(B)** Dose-response curve of radicicol against *P. falciparum* Dd2 blood-stage parasites. **(C)** Dose-response curve of radicicol against *P. berghei* liver-stage parasites. **(D)** Cytotoxicity assessment of radicicol in HepG2 human hepatocytes.

bioRxiv preprint doi: https://doi.org/10.1101/2025.10.30.684657; this version posted November 1, 2025. The copyright holder for this preprint (which was not certified by peer review) is the author/funder, who has granted bioRxiv a license to display the preprint in perpetuity. It is made available under aCC-BY 4.0 International license.

830 **Figure S2**

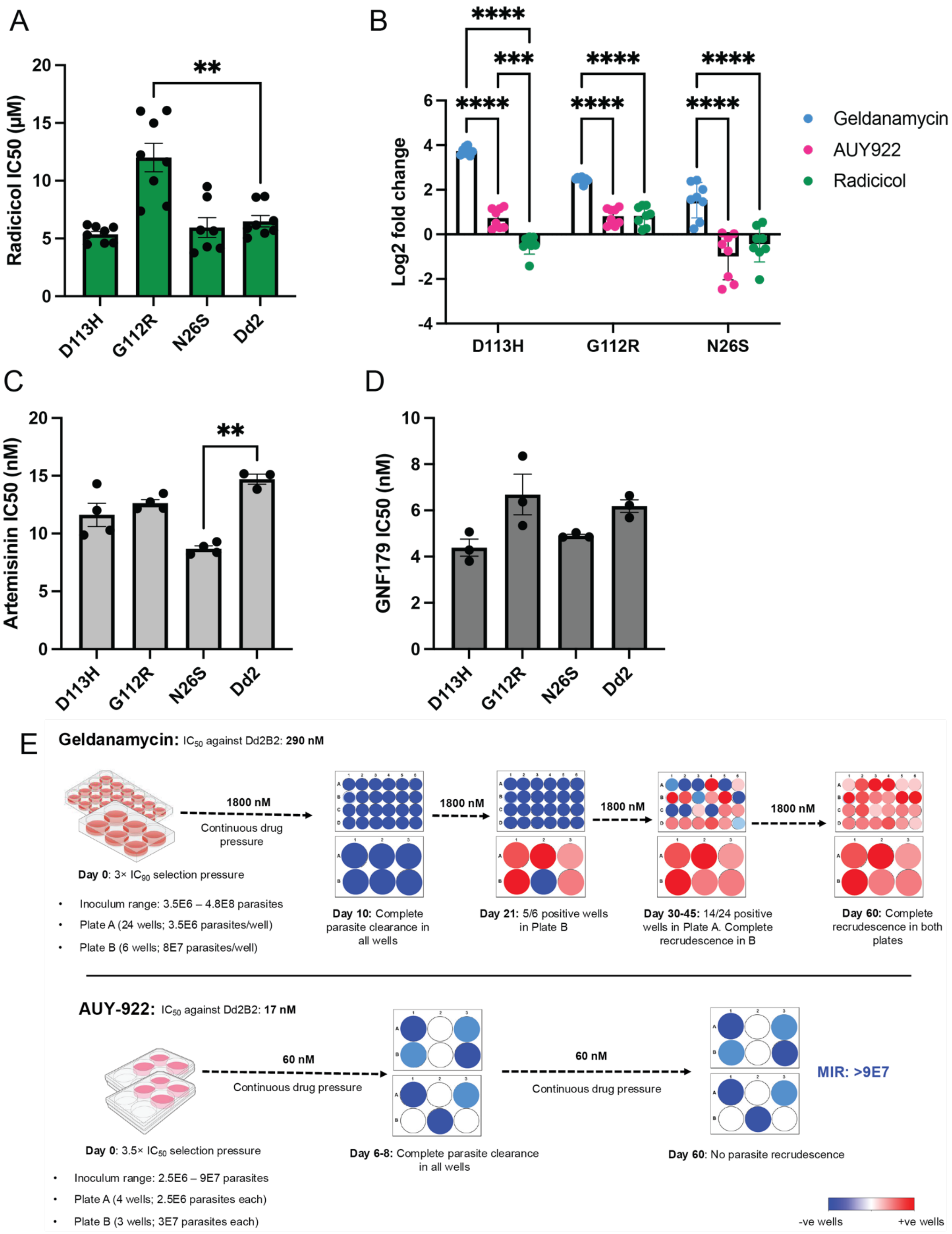

831

bioRxiv preprint doi: https://doi.org/10.1101/2025.10.30.684657; this version posted November 1, 2025. The copyright holder for this preprint (which was not certified by peer review) is the author/funder, who has granted bioRxiv a license to display the preprint in perpetuity. It is made available under aCC-BY 4.0 International license.

Figure S2. Cross-resistance characterization of geldanamycin-resistant clones and Minimum Inoculum for Resistance analysis

(A) IC_{50} values of geldanamycin-resistant clones against radicicol. (B) Log_2 fold-change analysis of geldanamycin-resistant mutations (D113H, G112R, N26S) against geldanamycin (blue), AUY-922 (magenta), and radicicol (green). **(C)**IC_{50} values of geldanamycin-resistant clones against artemisinin. **(D)** IC_{50} values of geldanamycin-resistant clones against GNF179. **(E)** Minimum Inoculum for Resistance (MIR) assay results. Top: Geldanamycin MIR assay progression from Day 0 to Day 60. Bottom: AUY-922 MIR assay showing no recrudescence (MIR $>9\times10^7$). Blue circles represent negative wells, red circles represent positive wells, white circles represent wells not tested.

bioRxiv preprint doi: https://doi.org/10.1101/2025.10.30.684657; this version posted November 1, 2025. The copyright holder for this preprint (which was not certified by peer review) is the author/funder, who has granted bioRxiv a license to display the preprint in perpetuity. It is made available under aCC-BY 4.0 International license.

Figure S3

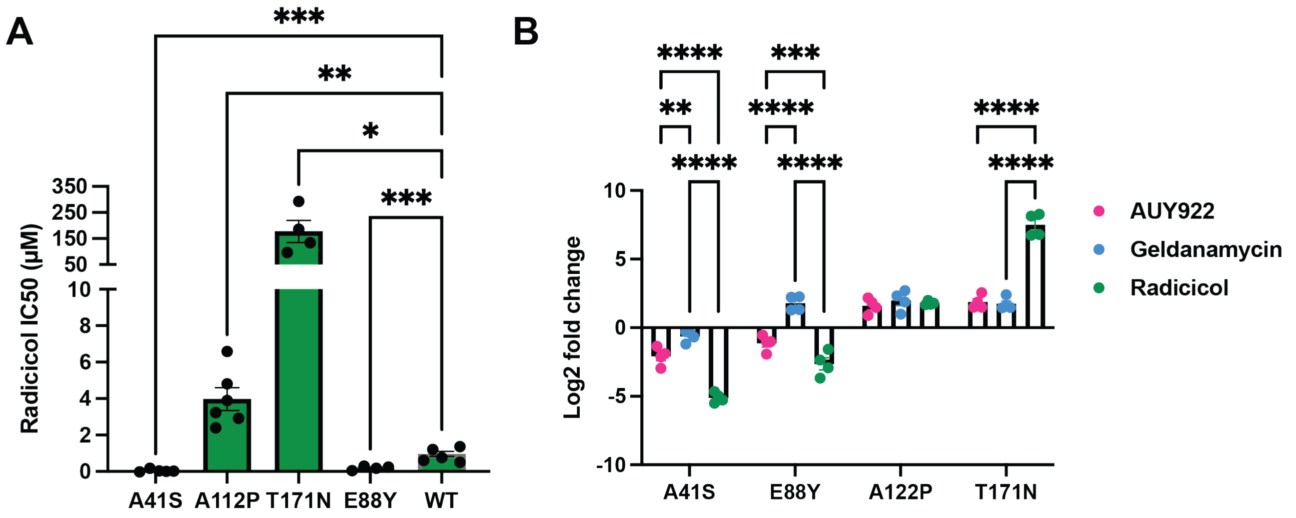

Figure S3. Cross-species comparison of HSP90 inhibitor sensitivity in *S. cerevisiae*

(A) IC_{50} values of CRISPR-engineered *S. cerevisiae* HSP90 mutations against radicicol. **(B)** IC_{50} fold-changes of CRISPR-engineered *S. cerevisiae* HSP90 mutations against AUY-922, geldanamycin, and radicicol, demonstrating mutation-specific resistance and sensitivity patterns across the three HSP90 inhibitors.

bioRxiv preprint doi: https://doi.org/10.1101/2025.10.30.684657; this version posted November 1, 2025. The copyright holder for this preprint (which was not certified by peer review) is the author/funder, who has granted bioRxiv a license to display the preprint in perpetuity. It is made available under aCC-BY 4.0 International license.

Figure S4

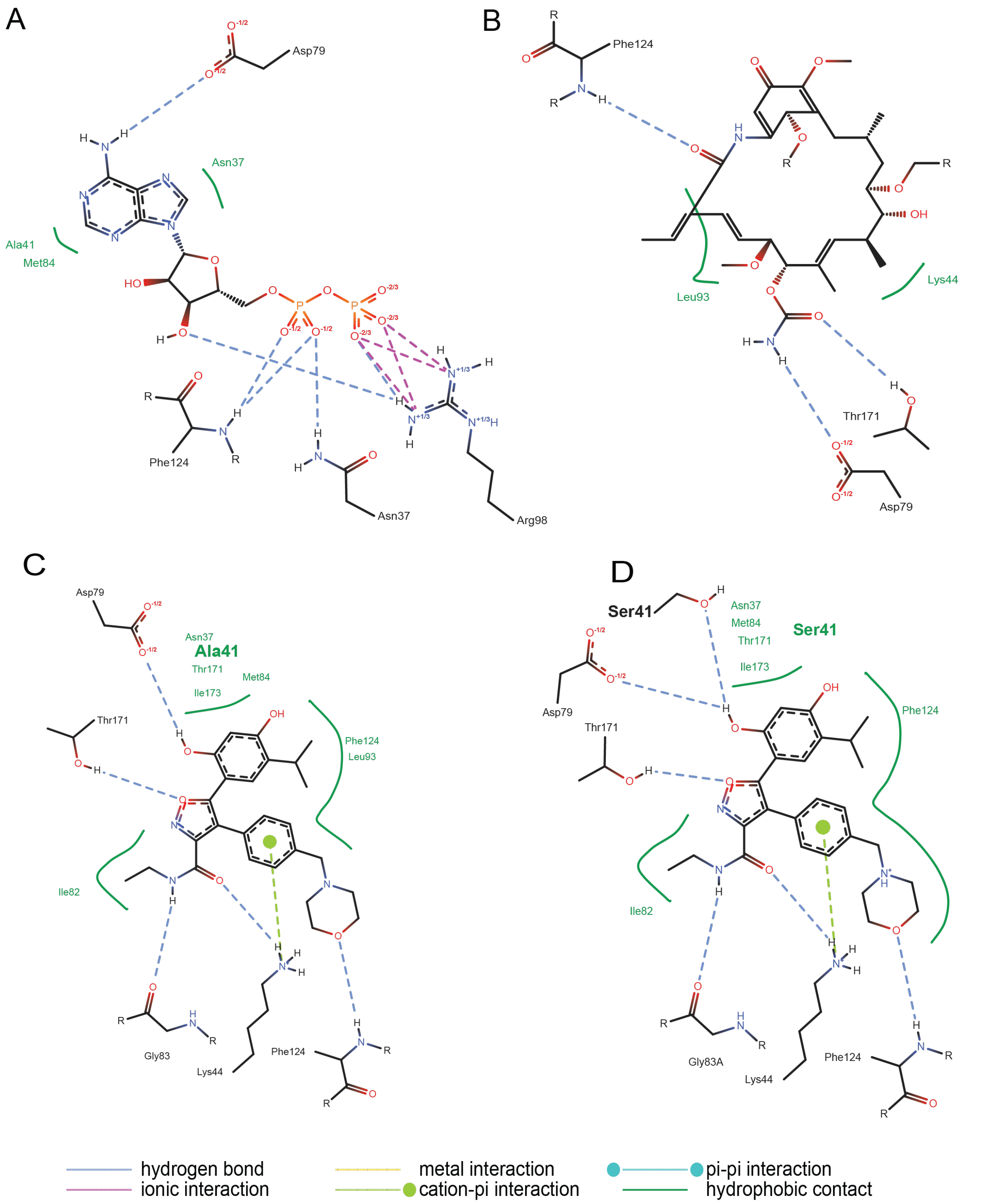

Figure S4. PoseEdit ligand interaction analysis

bioRxiv preprint doi: https://doi.org/10.1101/2025.10.30.684657; this version posted November 1, 2025. The copyright holder for this preprint (which was not certified by peer review) is the author/funder, who has granted bioRxiv a license to display the preprint in perpetuity. It is made available under aCC-BY 4.0 International license.

(A) PoseEdit interaction diagram of ADP binding to PfHSP90. **(B)** PoseEdit interaction diagram of geldanamycin binding to PfHSP90. **(C)** PoseEdit interaction diagram of AUY-922 binding to wild-type PfHSP90 with Ala41 highlighted. **(D)**PoseEdit interaction diagram of AUY-922 binding to PfHSP90 A41S mutant with Ser41 highlighted.

bioRxiv preprint doi: https://doi.org/10.1101/2025.10.30.684657; this version posted November 1, 2025. The copyright holder for this preprint (which was not certified by peer review) is the author/funder, who has granted bioRxiv a license to display the preprint in perpetuity. It is made available under aCC-BY 4.0 International license.

Figure S5

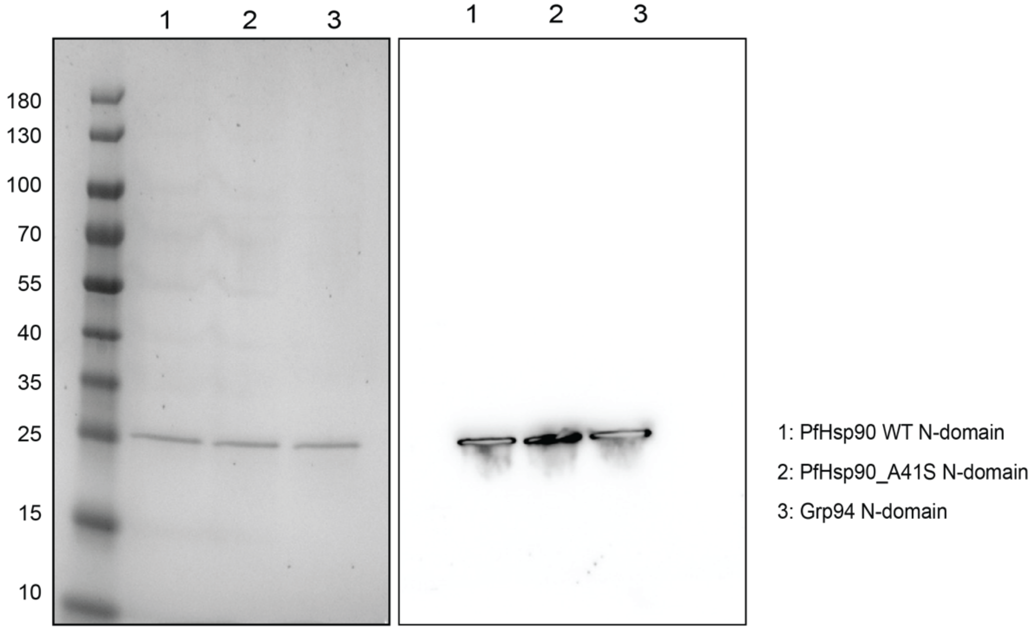

Figure S5. Protein purification validation

SDS-PAGE analysis showing purified proteins. Left: Coomassie blue staining. Right: Anti-His Western blot confirming successful purification of PfHsp90 wild-type N-domain, PfHSP90 A41S N-domain, and PfGRP94 N-domain.

bioRxiv preprint doi: https://doi.org/10.1101/2025.10.30.684657; this version posted November 1, 2025. The copyright holder for this preprint (which was not certified by peer review) is the author/funder, who has granted bioRxiv a license to display the preprint in perpetuity. It is made available under aCC-BY 4.0 International license.

Figure S6

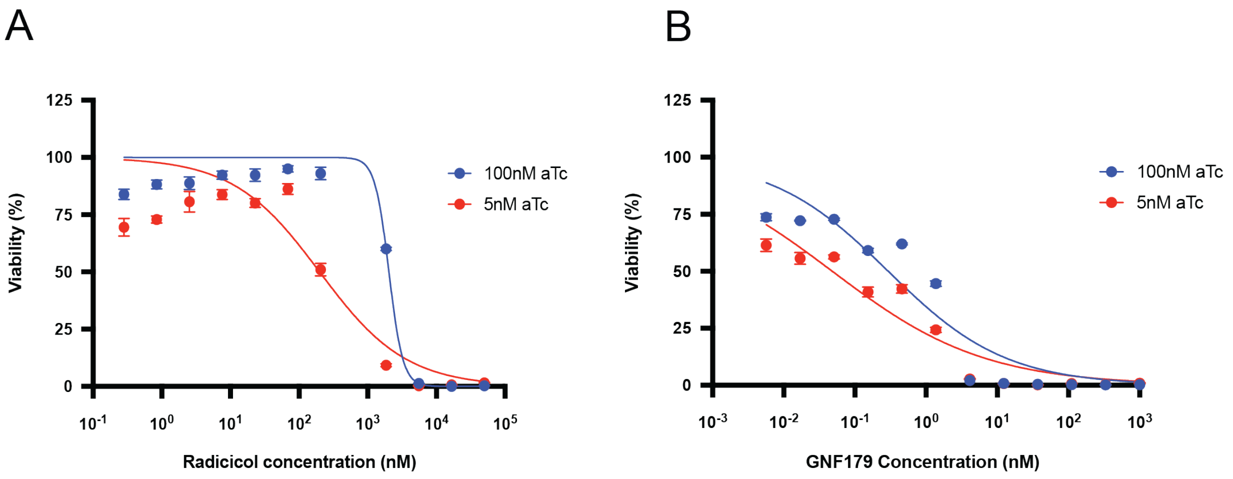

Figure S6. HSP90 conditional knockdown effects on radicicol and GNF179 sensitivity

(A) IC_{50} shift assay for radicicol under HSP90 knockdown conditions comparing 5 nM vs 100 nM aTc treatment. (B) IC_{50} shift assay for GNF179 under HSP90 knockdown conditions comparing 5 nM vs 100 nM aTc treatment.
